# From Correlational Signs to Markers. Current Trends in Neuroelectric Research on Visual Attentional Processing

**DOI:** 10.3390/brainsci10060350

**Published:** 2020-06-06

**Authors:** Alberto Zani

**Affiliations:** School of Psychology, Vita Salute San Raffaele University, 20132 Milan, Italy; zani.alberto@hsr.it

**Keywords:** EEG, ERPs, brain visual attentional processing, neural markers, intracerebral single and distributed electric source localization analyses, hemodynamic imaging, psychological sciences, cognitive neurosciences

## Abstract

Traditionally, electroencephalographic (EEG) and event-related brain potentials (ERPs) research on visual attentional processing attempted to account for mental processes in conceptual terms without reference to the way in which they were physically realized by the anatomical structures and physiological processes of the human brain. The brain science level of analysis, in contrast, attempted to explain the brain as an information processing system and to explain mental events in terms of brain processes. Somehow overcoming the separation between the two abovementioned levels of analysis, the cognitive neuroscience level considered how information was represented and processed in the brain. Neurofunctional processing takes place in a fraction of a second. Hence, the very high time resolution and the reliable sensitivity of EEG and ERPs in detecting fast functional changes in brain activity provided advantages over hemodynamic imaging techniques such as positron emission tomography (PET) or functional magnetic resonance imaging (fMRI), as well as over behavioral measures. However, volume conduction and lack of three-dimensionality limited applications of EEG and ERPs per se more than hemodynamic techniques for revealing locations in which brain processing occurs. These limits could only be overcome by subtraction methods for isolating attentional effects that might endure over time in EEG and may be riding even over several different ERP components, and by intracerebral single and distributed electric source analyses as well as the combining of these signals with high-spatial resolution hemodynamic signals (fMRI), both in healthy individuals and clinical patients. In my view, the articles of the Special Issue concerned with “ERP and EEG Markers of Brain Visual Attentional Processing” of the present journal Brain Sciences provide very good examples of all these levels of analysis.

## 1. Introduction

During the call for papers for the Special Issue concerned with “ERP and EEG Markers of Brain Visual Attentional Processing” of the present journal, I found myself compelled to move from the Institute of Molecular Bioimaging and Physiology (IBFM), where I spent about twenty years, to the Institute for the History of Philosophical and Scientific Thought in Modern Age (ISPF) of the Italian National Research Council (CNR), for a series of reasons that would be too long to explain here.

On my first days at the CNR-ISPF, some of my new theoretical philosopher colleagues asked me reasonable but at the same time “thorny” questions about my electrophysiological experimental research.

They asked me to characterize my electroencephalographic (EEG) and event-related brain potentials (ERPs) research as a “research program”, not from the perspective of a series of isolated studies but as a body of work which, over a period of ten or twenty years, has made definite progress in theory and methodology. What was my research program? What questions was I asking? What techniques were available to answer these questions? Could these techniques, in principle, provide the answers?

Fortunately, I had already dealt with such matters in the past in my editorial endeavors, e.g., [1,2]. Therefore, taking those questions as a challenge to myself, I tried to answer them later on explicitly during a presentation seminar, after some meditations to update and focus my thoughts on these matters.

I started my presentation by telling them that, for me, the truly big questions concerned human mental processes—among them, most specifically, visual selective attention processing e.g., [1,3,4,5,6], which I have particularly fancied over many years of my career—and their relationships to overt behavior. I also told them that, historically, these questions had been addressed at three different levels of analysis, which I referred to as the mind (psychological or cognitive sciences), the brain (brain science), and the cognitive neuroscience levels of analysis, very much aware that I might be somehow violating the common usage of these terms.

I went on to explain that, traditionally, the mind level attempted to account for mental processes in conceptual terms without reference to the way in which they were physically realized by the anatomical structures and physiological processes of the human brain [1,7,8,9]. Psychological theories in this sense did not use terms such as “superior parietal cortex” or “temporo-parietal junction” of the brain in relation to visuospatial attention orienting processes. Rather, they used other constructs, such as “visuospatial endogenous attention orienting” and/or “visuospatial exogenous attention orienting”, which, traditionally, did not imply any specific physical realization.

The brain science level of analysis, in contrast, attempted to explain the brain as an information processing system and to explain mental events in terms of brain processes. As such, it allowed the development of particular explanations, such as “visuospatial attention orienting consists of computational processes of neuronal circuits located in fronto-parietal and occipito-temporo-parietal structures of the brain having properties of engagement, disengagement of attention orienting and selection of relevant in comparison to irrelevant information [10,11,12].”

Somehow overcoming the separation between the two abovementioned levels of analysis, the cognitive neuroscience level—which I explained I had enthusiastically embraced for my endeavors for quite some time—considered how information was represented and processed in the brain, i.e., the mental/psychological level. At the same time, an analysis at the cognitive neuroscience level allowed one to pursue the exploration of underlying functional organization of distributed but often overlapping brain networks involved as a set of algorithms in mental functions in general and in visuospatial attentional functions in particular, i.e., the brain/neuropsychological level of analysis. From this perspective, every statement about mind/psychology was a statement about brain function [12,13,14].

Now, where did EEG and ERP research fit into this characterization? EEG and ERP are continuous, multidimensional electrical signal waveforms reflecting brain voltage fluctuations in time. These waveforms consist of a series of positive and negative electrical voltage deflections relative to baseline activity [1,2,7,9,15,16,17,18,19,20]. Only those voltage deflections varying as a function of the changes in the flow of information during cognitive processing are indicated as intervening EEG spectra or ERPs “components”—or “scalp manifestations”—of the abovementioned changes [1,2,7,17].

Neurofunctional processing takes place in a fraction of a second. Hence, the very high time resolution—in the order of milliseconds—and the reliable sensitivity of EEG and ERP in detecting functional changes in brain activity as well as the noninvasiveness of these techniques provided advantages [15,16,17,18,19] over hemodynamic imaging techniques such as positron emission tomography (PET) or functional magnetic resonance imaging (fMRI), as well as over behavioral measures [12,13], for investigating details of brain functional organization and the timing of the activation of regional areas of anatomically distributed processing systems involved in cognitive functions [1,2].

It is true that volume conduction and the lack of three-dimensionality limit applications of EEG and ERPs per se more than hemodynamic techniques for revealing locations in which brain processing occurs [1,2,7,9,14]. A limitation much less attributable to magnetoencephalography (MEG), the complementary magnetic signals that simultaneously accompany EEG and ERP signals, which should be interpreted together whenever possible [21,22]. However, simply knowing the neural locus of a given functional activation can in no way explain the mechanisms of a cognitive domain, such as how we direct our attention to objects and the surrounding space [1,23,24,25,26].

I worked to explain that at the cognitive neuroscience level of analysis, the neural localization of mental domains was considered only as good as the unveiling of the mechanisms underlying the different mental domains [23,24,25,26], such as visuospatial attention processing [1,2,20].

Therefore, while relating variations in EEG and ERPs to changes in psychological processes is important for adding knowledge of the mechanisms governing mental domains, it cannot by itself contribute to knowledge at the brain localization level [1,2,7,9,17]. As a consequence, to cope with the spatiotemporal overlap in the scalp-recorded bioelectrical manifestations of underlying single brain structure activation and with problems in determining their neurophysiological generators, cognitive electrophysiologists identify the portions of the recorded waveforms that can be independently changed by different cognitive conditions as true components of activity in specific cerebral structures. These components can only be identified by subtraction methods for isolating effects that might endure over time and may be riding even over several different components. Importantly, these subtraction methods can be applied to investigate attention effects (i.e., the differential brain response to items that are attended with respect to those that are not) [1,2,7,17].

With the theoretical backing of modern cognitive neuroscience, high-time resolution EEG and ERP research has been augmented by new developments at a dizzying speed over recent years. This occurred thanks to the introduction of new techniques, e.g., Morlet’s wavelet (the time-related EEG band power-spectra computation [2,16,23]) and algorithms for intracerebral single source analyses such as Brain Electrical Source Analysis (BESA) [1,2,14,27], and, later on, for distributed intracerebral source localization analyses such as Low Resolution Tomography (LORETA) [28]. In its upgraded versions, i.e., exact LORETA (eLORETA) or sLORETA and (swLORETA), the algorithm assumes that extended segments of the brain cortex can be active simultaneously while allowing the contribution of single areas to vary over time [1,2,29,30]. Indeed, thanks to the introduction of these new techniques and of the constrained “combining” with hemodynamic methods (e.g., MRI, fMRI), EEG and ERPs have contributed, as a research tool, to both cognitive and brain sciences, putting together new knowledge about humans as integrated sociobiological individuals [1,2,11,12,17,20,31,32].

These contributions also occurred thanks to the application of EEG and ERPs in the evaluation of psychological and psychiatric as well as neurological patients and to reciprocal relationships between the clinic and the experimental laboratory in the framework of an interdisciplinary integration of neurofunctional concepts and cognitive protocols and models, such as those proposed in cognitive neuroscience [33,34,35]. With hindsight, I indicated to my new colleagues these contributions and relationships as being specially relevant for EEG and ERP research, although, in my entire career, I rather rarely worked with patients and/or clinical populations (e.g., see the “References” section for my sober “contributions” to the literature [36,37]). These studies, in fact, not only allow the probing of brain processing when the system is impaired but also the involvement of localized brain districts in cognitive domains, as is done by traditional clinical neuropsychology for the benefits of patients. Most importantly, they shed some light on neural sources of ERP components possibly involved in cognitive processing [1,2,33], as directly pursued in the past by means of intracerebral microelectrode recordings during surgery in human patients [38] and brain lesional studies in animals to the detriment of fair ethical judgments.

I concluded my presentation by stating that I believed that cognitive electrophysiology had at present laid the groundwork for the use of EEG and ERPs, in combination with other available techniques and analysis methods, as quantifiable “markers” of integrated cognitive and affective processing of the brain. I added that, using these markers, during my career, I had been humbly able, together with my colleague Alice Mado Proverbio and our coworkers, to make some contributions to the testing of existing general theories on the mind and brain, especially with respect to visuospatial attentional processing [1,2,10], and, perhaps, to advancing fresher and more heuristic ones [39,40,41]. I also outlined that to be successful in this endeavor of exploring how visual attention processing arose from specific brain structures, I had worked in the context of interdisciplinary cognitive and brain functional theories as well as with sophisticated methodologies.

I have used these reflections to guide my work when, unexpectedly, after a few months spent at the CNR-ISPF, I was, for better or for worse, hired as a full professor of general and experimental psychology from the School of Psychology of Vita-Salute San Raffaele University. Indeed, it is also in the framework of the theoretical assumptions and “research program” discussed above that I gave my final evaluations of the reviewing process as a guest editor on the influential papers published in the Special Issue, out of the several others submitted.

## 2. The “ERP and EEG Markers of Brain Visual Attentional Processing” Special Issue—Overview

The research articles of this Special Issue—published by a panel of authoritative international cognitive neuroscientists and electrophysiologists—present state-of-the art developments in the knowledge of the relationships between visuospatial attentional processing and the brain as investigated by means of EEG and ERPs in the framework of theoretical orientations of cognitive science and neuroscience. All the articles compare overt behavioral data obtained in universally renowned visual selective attention protocols with the electrophysiological data obtained in these same protocols aimed at investigating different facets of visuospatial attentional processing. The articles have been published by the *Brain Sciences* journal at different dates during the call for papers for the Special Issue. Rather than following their publication order, I thought that it was appropriate to organize their presentation into relative theoretical and methodological as well as “thematic sections” in order to guarantee to each of them the deserved regards in the framework of a systematic discussion.

For the cognitive/psychological level of analysis of brain function “EEG markers” section, the article by Alberto Zani, Clara Tumminelli, and Alice Mado Proverbio [42] robustly proved that EEG alpha power computed during both a typical normoxia and an atypical oxygenation condition (hypoxia) in the brain might usefully serve as a marker of visuospatial attention orienting or suppression. Alpha power was lowest during the exogenous orienting of spatial attention, highest during alerting, and intermediate during the endogenous orienting of attention, regardless of brain oxygenation levels. The data also indicated that the dramatic increase in alpha power found in hypoxia over the right-sided lateral occipito-parietal scalp areas, independent of attention cueing and conflict conditions, possibly marked an overall suppression or impairment of separate visual attention network functionality.

Most interestingly, Emmanuelle Tognoli’s paper [43] also considered how EEG brainwaves within the 10 Hz band governed brain visuospatial attentional processing. At the same time, the author discussed theoretically the salient power distribution and privileged timescale of these waves for cognition and behavior, hinting positively at the intriguing possibility that a number of other 10 Hz neuromarkers had function and topography clearly distinct from alpha power, such as an activity measurement named *xi* (ꭓ), recorded over the left centroparietal scalp regions when subjects held their attention to spatially peripheral locations while maintaining their gaze centrally. The author challengingly outlines several potential functions for *xi* (ꭓ) activity as a putative neuromarker of covert attention distinct from alpha power.

Both of the abovementioned papers provide the foremost constraints for further probing the functions of these 10 Hz brainwaves in close relation to brain neuroanatomy as searched by combining these brainwaves with hemodynamic and/or single and distributed source modeling data.

Still at the cognitive/psychological level of analysis, Claudio de’Sperati’s paper [44] measured steady-state visual evoked potentials (SSVEPs) to video speed modulations in a group of observers and found a clear perceptual sensitivity increase and a moderate SSVEP amplitude increase with increasing speed modulation strength. Importantly, cortical responses also appeared with weak, overtly undetected speed modulations. Overall, these preliminary ERP findings suggest that the brain cortex responds selectively to periodic stimulus speed modulations, even when observers are not aware of them, thus hinting at an entrainment mechanism that may be the basis of a perceptual automatic resonance to the rhythms of the external visual world.

Further contributing to the cognitive/psychological level of study of brain functions, Tugba Kapanci’s, Sarah Merks’s, Thomas H. Rammsayer’s, and Stefan J. Troche’s study [45] illustrated how latency measurements of ERP P3 recorded in an increasing demand selective attention task administered to a sizeable sample of participants allowed them to settle the inconsistency in the literature of findings on the chronometric speed of information processing as a function of mental ability (MA), with higher speed in individuals with higher MA vs. the speed of those with lower MA. Indeed, using this increasing demand task, the authors were able to show that, in agreement with overt behavioral data (RTs) increases, MA and P3 latency negatively correlated in the standard condition of a continuous performance test (CPT), and this negative relationship increased systematically from the higher to the highest task demand conditions (CPT1 vs. CPT2) but was absent in the lowest demand (CPT0) condition.

To test how the brain visual attentional processing system dealt with changes in the relevance of stimulus features in time and space, Rolf Verleger, Kamila Śmigasiewicz, Lars Michael, Laura Heikaus, and Michael Niedeggen [46] used a task with rapid serial presentation of two stimulus streams where two targets (“T1” and “T2”) had to be distinguished from background stimuli and where the difficult T2 distinction was impeded by background stimuli presented before T1 that resembled T2 (“lures”). A blue digit among black letters was used as T2, and lures resembled T2 either by alphanumeric category (black digits) or by salience stimuli (blue letters). Same-category lures were expected to prime T2 identification, whereas salient lures would impede T2 identification. Behavioral results confirmed these predictions, yet the precise pattern of results did not fully fit the authors’ conceptual framework. Selection mechanisms were additionally probed by measuring ERPs. Consistent with the assumption that color lures attracted more attention, they induced larger N2pc than digit lures and affected the ensuing T1-related N2pc. T2-evoked N2pc was indistinguishably reduced by all kinds of preceding lures. A lure-related enhancement of mesio-frontal negativity from the first lure to the third lure both with digit and color lures also hinted at an increase in expectancy for T1.

Most interestingly, three articles gathered in a “clinical section” of the simultaneous psychological and brain levels of analysis provide new insights on the use of ERPs in both psychological and neurological clinical settings and into the fruitful, reciprocal relationships between the clinic and the experimental laboratory, thanks to the addition of cognitive neuroscience complex protocols to the traditional clinical ones.

The study by Manon E. Jaquerod, Sarah K. Mesrobian, Alessandro E. P. Villa, Michel Bader, and Alessandra Lintas [47] provided robust data showing that Working Memory training (WMT) with a high cognitive load affected the top-down modulation of brain visual attentional processing in a probabilistic gambling task in young Attention Deficit Hyperactivity Disorder (ADHD) adults compared with matched controls. ERPs elicited by the choice of the amount wagered in a gambling task were recorded before and after WMT in a dual n-back task in these two groups. In ADHD, the P1 wave component was selectively affected at frontal sites, and its shape was recovered close to that of controls only after adaptive WMT. Based on these findings, they propose that modified frontal site activities might constitute a neural marker of this effect in a gambling task. In controls, conversely, an increase in late parietal negativity might rather be a marker of an increase in transfer effects to fluid intelligence.

Yanni Liu, Gregory L. Hanna, Barbara S. Hanna, Haley E. Rough, Paul D. Arnold, and William J. Gehring [48] provided behavioral and ERP component data positively contributing to closing the gap in knowledge concerning the developmental trajectory of brain visual attentional processing underlying performance deficits in children and adolescent youths with ADHD. Compared with healthy controls (HCs), participants with ADHD obtained slower and more variable reaction times (RT), as well as reduced post-error slowing, in a congruent–incongruent arrow flankers task; they also exhibited reduced error-related negativity (ERN) and error positivity effects and reduced N2 and P3 congruency effects. Most importantly, developmental effects were observed across groups: with increasing age, participants responded faster, with less variability, and with increased post-error slowing. They also exhibited increased ERN effects and increased N2 and P3 congruency effects. Increased variability in RTs and reduced P3 amplitudes in incongruent trials were associated with increased ADHD Problems Scale scores on the Child Behavior Checklist across groups.

The study by Julie Bolduc-Teasdale, Pierre Jolicoeur, and Michelle McKerral [49] adds original findings on different forms of mild traumatic brain injury (mTBI) compared to uninjured controls by means of a novel and sensitive as well as rigorous ERP task implemented at the diagnostic and follow-up levels. Thanks to this task, the authors were able to show that both earlier VEPs (P1, N1) and visuospatial-attention orienting-related N2pc as well as encoding in visual short-term memory (vSTM), at later stages of brain processing, were as a whole comparable between mTBI and controls. However, there appeared to be a disruption in the spatiotemporal dynamics of attention (N2pc-Ptc, P2) in subacute mTBI that recovered within six months. This pattern was also reflected in altered neuropsychological performance (information processing speed, attentional shifting). Most interestingly, the orientation of attention (P3a) and working memory processes (P3b) were also affected and remained as such in the chronic postmTBI period, in cooccurrence with persisting post-concussion symptomatology.

Last but not least, a small collection of three additional papers also directly assessed ERP manifestations of visual attentional processing with their brain area localizations either with both single dipole (e.g., D’Angiulli et al.) [50] and distributed swLORETA source modeling (e.g., Orlandi and Proverbio) [51] or with hemodynamic fMRI data (e.g., Morgan et al.) [52].

The study by Amedeo D′Angiulli, Dao Anh Thu Pham, Gerry Leisman, and Gary Goldfield [50] sought to examine enhanced brain responses to targets during visual sustained selective-set attention in preschool children. Notably, the study indicated conceivable novel directions for further tests and falsifiable hypotheses on the origins and development of visual selective attention brain networks and their ERP manifestations. ERPs concurrent with target presentation were, in fact, enhanced relative to distractors, without manual response confounds. Triangulation of peak analysis, ERP-based adaptive control of thought–rational (ACT–R) modeling, and simulation for the entire ERP epochs up to the moment of manual response (~700 ms, on average) suggested converging evidence of distinct but interacting processes of enhancement and planning for response release/inhibition, respectively. The latter involved functions and structures consistent with adult ERP activity, which might correspond to a large-scale network involving dorsal and ventral attention networks, corticostriatal loops, and subcortical hubs connected to prefrontal cortex top-down working memory executive control.

Further emphasizing ERP-related research on brain networks subserving visual attention, the study by Andrea Orlandi and Alice Mado Proverbio [51] provided evidence on the time course and activity in the left hemisphere of the brain underlying object-based attention. They recorded both the behavioral and ERP responses to 3D graphic images falling into different object categories (e.g., wooden dummies, chairs, structures of cubes) posing alternatively as targets and nontargets in separate runs of a selection task. Nontarget stimuli elicited a larger anterior N2 component, which was likely associated with motor inhibition. Conversely, target selection resulted in an enhanced selection negativity (SN) response lateralized over the left occipito-temporal regions, followed by a larger centro-parietal P300 response. These potentials were interpreted as indexing attentional selection and categorization processes, respectively. The left-sided generators of SN were also supported by distributed standardized-weighted low-resolution electromagnetic tomography (swLORETA) source reconstruction, which indicated a fronto-temporo-limbic network as a system directly involved in object-based visual attention.

Importantly, the multi-experiment study by Kyle K. Morgan, Dagmar Zeithamova, Phan Luu, and Don Tucker [52] used both fMRI and ERP measures to determine whether multiple memory systems are involved in a categorization task and to evaluate the time course under which these systems are recruited. Their findings robustly showed that once the participants acquired the task, clear differences in the left lateral inferior anterior negativity (LIAN), medial frontal negativity (MFN), and P3b components were seen between visually similar and visually distinct category conditions. A region-based fMRI multivoxel pattern analysis (MVPA) also showed that the lateral frontal and parietal regions provided the most reliable classification between the visual categories, consistent with previous findings that rule-based categorization requires a higher degree of attentional resources. Additionally, based on the correspondence of MFN latency with the initial orienting of attention in a visuomotor association task, the authors propose that the MFN is indexing the controlled attention allocated to select the memory system best suited for categorizing the perceived stimulus.

As a concluding remark, I would add that, in my view, this Special Issue represents a further step in the disclosure of “the principles to which brain areas are assigned to functions and get assembled into circuits [20]” as well as in networks, whose dynamic activations during visual selective processing may be directly and robustly reflected by EEG and ERP signals, as well as their MEG counterpart.
